# Nanoscale thickness Octave-spanning coherent supercontinuum light generation

**DOI:** 10.1038/s41377-024-01660-6

**Published:** 2025-01-09

**Authors:** Susobhan Das, Md Gius Uddin, Diao Li, Yadong Wang, Yunyun Dai, Juha Toivonen, Hao Hong, Kaihui Liu, Zhipei Sun

**Affiliations:** 1 Department of Electronics and Nanoengineering, Aalto University, Espoo, Finland; 2 QTF Centre of Excellence, Department of Applied Physics, Aalto University, Aalto, Finland; 3 Department of Engineering and Natural Sciences, Tampere University, Tampere, Finland; 4 State Key Laboratory for Mesoscopic Physics and Frontiers Science Center for Nano-optoelectronics, School of Physics, Peking University, Beijing, China

**Keywords:** Nonlinear optics, Supercontinuum generation

## Abstract

Coherent broadband light generation has attracted massive attention due to its numerous applications ranging from metrology, sensing, and imaging to communication. In general, spectral broadening is realized via third-order and higher-order nonlinear optical processes (e.g., self-phase modulation, Raman transition, four-wave mixing, multiwave mixing), which are typically weak and thus require a long interaction length and the phase matching condition to enhance the efficient nonlinear light-matter interaction for broad-spectrum generation. Here, for the first time, we report octave-spanning coherent light generation at the nanometer scale enabled by a phase-matching-free frequency down-conversion process. Up to octave-spanning coherent light generation with a −40dB spectral width covering from ~565 to 1906 nm is demonstrated in discreate manner via difference-frequency generation, a second-order nonlinear process in gallium selenide and niobium oxide diiodide crystals at the 100-nanometer scale. Compared with conventional coherent broadband light sources based on bulk materials, our demonstration is ~5 orders of magnitude thinner and requires ~3 orders of magnitude lower excitation power. Our results open a new way to possibly create compact, versatile and integrated ultra-broadband light sources.

## Abstract (web summary)

The article introduces a new method for nanoscale octave-spanning coherent light generation via phase-matching-free down-conversion, offering high-efficiency and applications in metrology, spectroscopy, and telecommunications.

## Graphical abstract

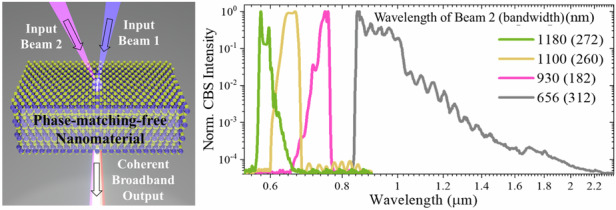

## Introduction

In nonlinear optics, spectral broadening and generation of new frequency components have been investigated for a long time since the invention of the laser in 1960. Among various coherent broadband light generation methods, supercontinuum (also called white-light continuum) generation^1–3^ represents a unique and versatile process to produce light with a spectrum over a considerable part of the visible and infrared spectrum simultaneously^4–6^, whereas other methods (for example, Raman transition, four-wave mixing, multiwave mixing, optical Kerr effect, high harmonic generation) offer discrete narrow spectrum separately to cover broadband^7–15^. The SC light sources are widely used for diverse applications, such as imaging^16^, metrology, communication, and LIDAR systems^17^. SC generation was first reported in bulk glasses^1,18^, and has since been the subject of numerous investigations in a wide variety of nonlinear media, including solids, liquids, and gases^19^. The current state-of-the-art SC sources mainly use third-order optical nonlinearity (e.g., self-phase modulation, four-wave mixing, and soliton effects). However, third-order optical nonlinearity susceptibility (χ^(3)^) usually is weak (e.g., 2.2 × 10^−22^ m^2^/V^2^ in silica^20^, 2.5 × 10^−19^ m^2^/V^2^ in silicon^21^). As a result, to achieve broad spectral output (e.g., >100 nm spectral coverage), SC sources typically require long waveguides (e.g., silica fibers^2,22–27^, silicon waveguides^28–33^) with different microstructures (e.g., photonic crystal fibers) and cascaded nonlinear optical processes to significantly enhance the light-matter interaction.

In general, for a noncentrosymmetric bulk medium, the magnitude of second-order optical nonlinear susceptibility (χ^(2)^) is ~8 to 10 orders of magnitude higher than the χ^(3)^^34^. Therefore, coherent broadband supercontinuum (CBS) generation based on second-order nonlinear optical processes potentially has various advantages, such as higher efficiency and lower excitation power^35–37^. Despite the advantages of χ^(2)^ based broadband light generation, the phase mismatching requirements in bulk crystals between input and output beams typically limit the output performance (e.g., spectral coverage and generation efficiency).

In this work, we report octave-spanning coherent light generation at the nanometer scale for the first time by utilizing a phase-matching-free second-order nonlinear optical process in thin gallium selenide (GaSe) and niobium oxide diiodide (NbOI_2_) crystals. Due to their deep-subwavelength thickness (~ 100 nm), which is much lower than the coherence length (~ 400 nm) of the incident beams, the second-order nonlinear optical process is phase-matching free. A broad spectrum covering from the visible to the near-infrared region is generated in GaSe (NbOI_2_) with an excitation power 2-order (3-order) of magnitude lower than the conventional SC generation in free-space bulk materials^38^. Our results open a new approach to generate versatile and efficient broadband coherent radiation for diverse applications.

## Results

**Fig. 1:**
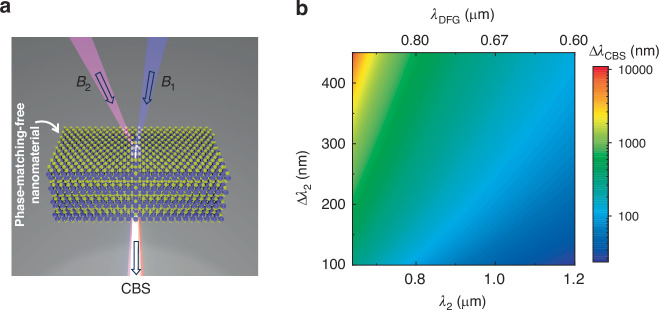
CBS generation principle with phase-matching-free second-order optical nonlinearity. **a** Schematic of CBS generation by a DFG process in a nanomaterial with a deep-subwavelength thickness. **b** The calculated output spectral width as a function of the central wavelength (λ_2_) of the input beam B_2_ and its spectral width ($\Delta$λ_2_) when λ_1_ is fixed at 400 nm

The schematic of the CBS generation process with a phase-matching-free second-order nonlinear process is shown in Fig. 1a, where two input light beams B_1_ (with a photon energy of *E*_*1*_) and B_2_ (with a photon energy of *E*_*2*_) generate a new light beam (with a photon energy of ${E}_{{CBB}}={E}_{1}-{E}_{2}$) via difference frequency generation (DFG). The maximum output spectral width ($\Delta$λ_CBS_) can be expressed as^34^

1 $${\Delta \lambda }_{{CBS}}=\frac{4{\lambda }_{1}^{2}{\Delta \lambda }_{2}}{4{\left({{\rm{\lambda }}}_{2}-{\lambda }_{1}\right)}^{2}-\Delta {\lambda }_{2}^{2}}$$

where λ_1_, λ_2_, and $\Delta$λ_2_ are the central wavelengths of the input beams B_1_ and B_2_, and the spectral width of B_2_, respectively. Here, we ignore the spectral width of the input beam B_1_ to simplify the discussion. A detailed explanation of Eq. 1 is provided in Supplementary Information.

The equation shows that the output spectral width highly depends on the input beam parameters. In principle, if the input beams are carefully chosen, the produced DFG beam spectrum can be correspondingly wide under a phase-matching-free condition. Figure 1b shows the calculated output spectral width when λ_1_ is fixed at 400 nm. It is evident that for a fixed bandwidth of the incident beam B_2_, the output bandwidth will be much broader (up to a few thousand nm) when the central wavelength λ_2_ is approaching λ_1_ due to the nature of the frequency down-conversion process. For example, when the input beam B_2_ has a central wavelength of 650 nm with a spectral width of 150 nm (e.g., the spectrum covers from ~575 to 725 nm), the output DFG spectrum can range from ~892 to 1314 nm with a central wavelength of 1103 nm and a spectral width of ~422 nm, which is ~3-times wider than that of the input beam B_2_.

Here, we choose the λ_1_ of 400 nm as an example because it is the second harmonic of the most widely used ultrafast Ti:sapphire laser. Other pump wavelength examples (e.g., λ_1_ = 355 or 532 nm, the harmonic wavelengths of the commonly used industrial solid-state and fiber lasers with Ytterbium- and Neodymium-doped gain materials) are shown in Supplementary Information. Nevertheless, with the DFG process under the phase-matching-free condition, it is possible to generate a broad spectrum with a careful design of the input conditions. Note that the concept can be applied to other parametric and non-parametric down-conversion processes based on higher-order optical nonlinearities (e.g., four-wave mixing in Supplementary information). However, higher-order ( > 2-order) optical nonlinearities are typically weaker than second-order. Therefore, for practical applications, we focus on the second-order optical nonlinearity-based DFG to demonstrate the proof of the concept, which can be further extended to other nonlinear optical processes.

To demonstrate the CBS generation concept, we use a ~ 88-nm thick GaSe flake as the nonlinear optical material. We also perform a similar experiment on thick NbOI_2_ flakes at the ~100-nm scale, which offer higher efficiency, as discussed later. Based on the SHG results at a pump wavelength of ~1200 nm, the calculated effective second-order nonlinear optical susceptibilities $\left|{\chi }_{{eff}}^{(2)}\right|$ are ~ 17.5 × 10^−11^ m/V and 9.034 × 10^−11^ m/V for NbOI_2_ and GaSe, respectively. For comparison, we also carry out similar experiments with monolayer MoS_2_, and a thick BBO crystal, yielding $\left|{\chi }_{{eff}}^{(2)}\right|$ of ~2.9 × 10^−11^ m/V and 1.6 × 10^−12^ m/V, respectively. Therefore, due to their high $\left|{\chi }_{{eff}}^{(2)}\right|$, we select NbOI_2_ and GaSe as the sample materials for our coherent broadband supercontinuum (CBS) demonstration. Our van der waals films have a thickness of ~ one order of magnitude smaller than the incident wavelengths and thus can fully enable various phase-matching-free nonlinear optical processes^39–47^. In our experiment, we tune the wavelength (λ_2_) of the input pulsed beam B_2_ with different spectral widths. Both incident pulsed beams are overlapped in the spatial and temporal domains to achieve efficient nonlinear optical processes. Details about the experimental setup, sample preparation and characterization are provided in Methods section and Supplementary information.

**Fig. 2:**
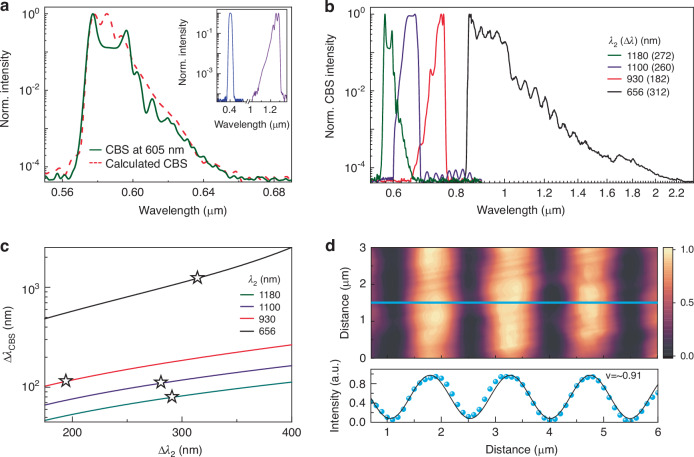
Nanoscale CBS demonstration. **a** Experimental (solid line) and theoretically calculated (dashed line) output CBS spectra. The two input light spectra are given in the inset. **b** Tunable CBS generation at different λ_2_ with the average power of ~10 µW (intensity ~153.42 GW/cm^2^). The spectral width ($\Delta$λ_2_) of the input beam B_2_ is given in the label with the central wavelength (λ_2_). λ_1_ = 400 nm with an average power of ~0.5 µW (intensity ~7.67 GW/cm^2^). **c** Calculated output spectral coverage (solid lines) with our experimental results (stars). **d** Coherence interference measurement (top) of the output centered at 650 nm and the corresponding fringe pattern at the position of the cyan line with sinusoidal fitting (bottom)

Figure 2a shows the generated CBS spectrum where the average incident powers of the input beams B_1_ and B_2_ are 0.5 µW (intensity ~7.67 GW/cm^2^) and 10 µW (intensity ~153.42 GW/cm^2^), respectively. The spectra of the input beams are shown in the inset of Fig. 2a. When the central wavelength of the incident beam B_2_ is at ~1180 nm with a spectral coverage of ~272 nm, we generate a ~ 72 nm wide spectrum at a central wavelength of ~605 nm. The generated CBS spectrum is highly correlated with both input beams and is firmly consistent with our theoretical calculation (dashed line in Fig. 2a) based on the envelope of the input beams. We further tune the wavelength of the input beam B_2_ with different spectral widths and get the corresponding output spectrum, as shown in Fig. 2b. The input conditions (i.e., the central wavelength of the input beam B_2_ (λ_2_) and the corresponding bandwidth ($\Delta$λ_2_)) are provided with it. When our input beam B_2_ is at ~656 nm with a spectral coverage of ~312 nm, the experimentally generated output spectrum spans from ~840 to 1906 nm with a −40 dB reference level, covering more than one octave. The corresponding bandwidth is ~1160 nm, close to 4 times wider than the input signal spectrum. Note that the output spectrum intensity at the longer wavelength is weaker (Fig. 2b), which is mainly caused by the decreased photodetector sensitivity (i.e., a wavelength range of ~350 and 2000 nm, details in Methods section) in the experiments. We highlight that, in the same experimental setup only via changing the input beam B_2_ parameters, the overall generated CBS spectrum covers the wavelength range from ~565 to ~1906 nm, spanning more than an octave with a −40 dB reference level. Please note that our output spectrum exhibits a small gap (~ 60 nm) at 800 nm. This is due to our selection of a 400 nm pump wavelength, which presents a challenge for generating a degenerate output at 800 nm. Nevertheless, we highlight that, in the same experimental setup only via changing the input beam B2 parameters, the overall generated CBS spectrum covers the wavelength range from ~565 to ~1906 nm. The generation of such broadband spectra at the nanometer scale mainly benefits from the efficient phase-matching-free strategy without any dispersion engineering. The theoretical calculations (solid lines) and the experimental results (stars) of the generated spectral coverage are shown in Fig. 2c, indicating a good agreement.

Output coherence of broadband light sources plays a key role in applications. Here, we measure the coherence of our CBS output (Setup details in Supplementary information). The interference fringe pattern of a Michelson interferometer of our CBS output is shown in Fig. 2d. Average fringe visibility is calculated above 0.9, indicating high coherence of the generated light^48^. The results are better than typical superluminescent light-emitting diodes (e.g., ~0.67^49^) and typical long-pulse pumped supercontinuum light sources whose coherence is generally lost[^50^.

The CBS output power from both GaSe and NbOI_2_ samples is measured as a function of the average power of both input beams. The slope of the linear fit of the CBS output power over both incident beam power is ~1 for both flakes (details in Supplementary information), which confirms that the DFG process is taking place in our experiment. We also observe the saturation of the generated CBS signal, which we attribute to the multi-photon absorption process of the input beam B_2_. We further optimize the experimental configuration and achieve ~9 nW with a larger GaSe flake and an enlarged beam spot size (~ 15 µm) when the pump powers *P*_*1*_ and *P*_*2*_ are ~20 µW (~ 8.52 GW/cm^2^) and ~150 µW (~ 63.92 GW/cm^2^), respectively.

**Fig. 3:**
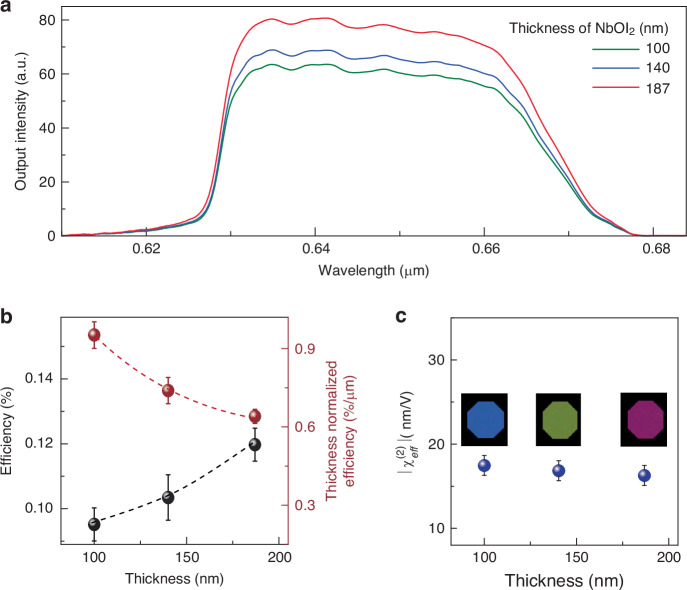
High conversion efficiency of nanoscale CBS with NbOI_2_. Thickness-dependent CBS spectra of (**a**) NbOI_2_ under the fixed input powers of ~0.1 µW (intensity ~1.53 GW/cm^2^) for both the input beams B_1_ and B_2_. **b** Thickness-dependent and thickness-normalized CBS conversion efficiency of NbOI_2_ flakes. **c** Calculated overall $\left|{\chi }_{{eff}}^{(2)}\right|$ of thin NbOI_2_ samples for different thicknesses. The optical images of the corresponding samples are shown in the inset

Since NbOI_2_ has a higher second-order nonlinear optical coefficient than GaSe, we use NbOI_2_ flakes^51^ to prove the generation efficiency can be improved if highly nonlinear optical materials or structures can be used. The results with NbOI_2_ are shown in Fig. 3a (output results with GaSe samples are in Supplementary information). Although the output CBS spectral envelopes look similar, thicker samples for both GaSe and NbOI_2_ materials give stronger signals under the same pump conditions as expected. The conversion efficiencies at different thicknesses are calculated by the average output power divided by the total power of the two input beams (black sphere, Fig. 3b), where thicker samples provide higher conversion efficiency.

We notice the saturation with a thicker GaSe sample mostly due to the unsatisfied phase-matching-free condition, which is evidenced by the slightly uneven output spectrum at ~314 nm sample thickness (Fig. S6 in Supplementary information). Therefore, a thicker sample is better for generating a strong CBS signal, but the thickness should not be larger than the coherence length (~ 400 nm) for generating flat spectra. As expected, the thicker sample has slightly lower effective second-order nonlinear susceptibility than the thin sample (Fig. 3c), but the variation is marginal. The calculated effective second-order susceptibility of NbOI_2_ is ~ 17.5 × 10^−11 ^m/V, which is consistent with the previously reported value^51^. The achieved maximum conversion efficiency for NbOI_2_ is ~0.12% (for GaSe is ~7.6 × 10^−3%^), which is one order of magnitude higher than the recently reported case with the thin film of aluminum-doped zinc oxide^52^. A table of comparison is provided in Supplementary information. Although, the maximum conversion efficiency achieved is relatively smaller when compared with the current state-of-the-art SC. However, given the nanometer thickness, the normalized conversion efficiencies are >~ 0.66^%^/µm for NbOI_2_ and ~0.025%/µm for GaSe (brown sphere in Fig. 3b and Figure S6b respectively), much higher than the previously reported free-space SC results^2,38^.

**Fig. 4:**
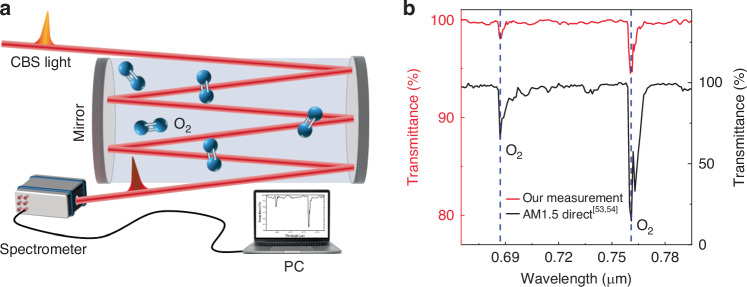
CBS application demonstration in gas sensing. **a** Schematic of gas sensing experimental setup, (**b**) the transmittance spectrum of oxygen measured with CBS and compared with references^53,54^

Our nanoscale broadband light source is promising for different applications, including spectroscopy, optical pumping, communication and gas sensing^38^. Here, we demonstrate its potential for gas sensing applications. With our CBS light source, we measure the transmittance spectrum of the room-air within the spectral window from ~650 nm to 800 nm, which is specifically the spectral absorption window for oxygen^53–55^]. After ~10 m free space propagation of generated CBS light (Fig. 4a), the obtained transmittance spectrum of oxygen is compared with the references^53,54^. The distinct transmission dips at ~687 nm and ~760 nm (blue dash lines in Fig. 4b) correspond to prominent oxygen absorption peaks assigned to collision pair absorption with an O_2_ excited to the upper state^55–57^.

Traditionally, spectral broadening or SC generation efficiency in a free-space bulk material usually relies on the optical material’s third-order optical susceptibility, which requires a strong incident power density and provides only a particular range of the optical spectrum. Further, the self-focusing effect is typically needed to increase the light-matter interaction for the SC generation. To initiate the self-focusing effect, the input power should be higher than the characteristic power, which is substantially large (e.g., pulse energy of ~40 nJ with peak power of ~0.27 MW for KGW, GdVO_4_, and YVO_4_ based bulk materials; pulse energy of ~172 nJ with peak power of ~1.15 MW for YAG crystals at 775 nm^38^). In contrast, our CBS generation with the phase-matching-free second-order optical nonlinear DFG method can take place with a much lower pulse energy of ~25 pJ (average power ~50nW, peak power ~108.5 W) for NbOI_2_ flakes and ~0.5 nJ (average power 1 µW, peak power of ~2.17 KW) for GaSe flakes, approximately 3 and 2-order of magnitude lower than the excitation threshold of the conventional free-space SC generation demonstrations respectively^38^. A table of comparison is provided in Supplementary information. Note that our samples at the nanometer scale are ~5-order of magnitude thinner than the existing free-space SC generation bulk crystals (e.g., 4 mm thickness for YAG crystals^38^). Further, based on the spectrum coverage by the input beams, we can tune the generated CBS spectrum, rather than selecting materials with proper dispersion engineering. Additionally, van der waals materials (such as GaSe, NbOI_2_ demonstrated here) have strong $\left|{\chi }^{(2)}\right|$ over a broad spectral range^40–46^. This enables efficient CBS generation at low pump power to cover more than one octave-spanning spectrum (Fig. 2b) in the visible and near-infrared regions.

In principle, the demonstrated concept can be extended to other phase-matching-free schemes (e.g., thin crystals, metasurfaces, epsilon-near-zero materials) based nonlinear optical processes (e.g., third- or higher-order optical nonlinear processes)^58–60^. For example, epsilon-near-zero devices based on low-dimensional materials (e.g., 1D, 2D materials^52^) with relatively large optical nonlinearities can effectively increase conversion efficiency. Further, phase-matching-free nonlinear optics has been recently demonstrated in waveguides^59^, which is very promising to further address the conversion efficiency of our concept via different structured waveguides (e.g., photonic crystal fibers^61^ or silicon-based waveguides) and then use only one input beam via a self-beam parametric process with fully-integrated platforms. The futuristic idea of the development of integrated CBS sources is described in Supplementary Information.

## Discussion

In conclusion, we have demonstrated that the phase-matching-free second-order nonlinear optical susceptibility can be utilized for CBS generation, which traditionally uses third-order nonlinear optical phenomenon for generating SC. Using GaSe as a nonlinear optical material with <~100 nm thickness, we achieved the octave-spanning spectrum coverage from ~565 to 1906 nm in discrete manner to avoid overlapping between input and output spectra. We would like to emphasize that the entire broadband spectrum can ideally also be generated in a single measurement if the two input beams participating in the DFG process interact noncolinearly. Since the broadband light is generated by the DFG process, the temporal and spatial coherency is preserved. Additionally, because the CBS generation is assisted by the second-order nonlinear optical susceptibility, which is ~8 to 10 orders of magnitude higher than the third-order nonlinear optical susceptibility, the second-order nonlinearity-based CBS generation is more efficient than the traditional SC generation approaches. In this case, the power requirement for the CBS generation is ~3 orders of magnitude lower than the conventional SC generation systems from bulk crystals. Our demonstration of CBS generation via the phase-matching-free down-conversion nonlinear optical processes can lead to a new way to generate efficient ultra-broadband coherent light sources for applications, such as metrology, spectroscopy, and LIDAR systems.

## Materials and methods

The schematic of the experimental setup is shown in Supplementary information. An optical parametric amplifier (Spectra-Physics, TOPAS) with a repetition rate of 2 kHz and pulse width of ~230 fs width was used to generate both the input beams. The input beam B_1_ at 400 nm was achieved by frequency doubling of the fundamental source of the optical parametric amplifier with a barium borate crystal. To generate the input beam B_2_, another beam from the same laser is focused on a 0.5-mm thick sapphire substrate to broaden the spectrum. After that, optical filters are used to tune the center wavelength and spectral width of the input beam B_2_. After passing through a time delay line, both the pump beams are spatially merged using a dichroic mirror, and the combined beam is focused onto the sample with an objective lens. Nikon CFI Plan Fluor 40× (NA 0.75) objective lens is used to get 2.5 µm spot size is. To achieve 15 µm spot size, we used Nikon CFI Plan Fluor 10× (NA 0.3) objective. Afterwards, the transmitted DFG signal is separated from both input beams by optical filters. The DFG signal is measured with a photomultiplier tube (with a working wavelength range from ~0.35 to 0.950 µm) or InGaAs photodetector (with an operating wavelength range from ~0.9 to 2 µm) following a monochromator (Andor 328i). We used GaSe and NbOI_2_ bulk crystals from 2D Semiconductor and exfoliated them first on a blue tape (Nitto). Afterwards, GaSe/NbOI_2_ flakes were transferred onto a 1-mm thick glass substrate employing Polydimethylsiloxane (PDMS)-based dry-transfer technique with a home-built setup to prepare clean samples. An atomic force microscope measures the sample thicknesses at different positions. Micro-Raman is used to characterize all GaSe and NbOI_2_ flakes with different thicknesses, which confirms their high quality.

## Supplementary information

Supplementary Information: Nanoscale thickness Octave-spanning Coherent Supercontinuum Light Generation

## Data Availability

All data needed to evaluate the findings of this study are available within the Article and Supplementary Information. Source Data files are also available upon the request.
