# Supplementary material for: Nanoscale thickness Octave-spanning coherent supercontinuum light generation
Source: Light Sci Appl. 2025 Jan 9;14:41. doi: 10.1038/s41377-024-01660-6 (PMC11711750; doi:10.1038/s41377-024-01660-6)
Supplement: Supplementary file 1 — Supplementary Information: Nanoscale thickness Octave-spanning Coherent Supercontinuum Light Generation [file 41377_2024_1660_MOESM1_ESM.pdf]

Supplementary Information

Nanoscale thickness Octave-spanning Coherent Supercontinuum Light Generation

Susobhan Das[*1,2], Md Gius Uddin*[1,2], Diao Li*[1], Yadong Wang*[1], Yunyun Dai*[1], Juha Toivonen*[3], Hao Hong*[4], Kaihui Liu*[4], Zhipei Sun* [*1,2]

[1]Department of Electronics and Nanoengineering, Aalto University, Tietotie 3, FI-02150, Finland.
[2]QTF Centre of Excellence, Department of Applied Physics, Aalto University, Aalto FI-00076, Finland
[3]Department of Engineering and Natural Sciences, Tampere University, Tampere-33720, Finland
[4]State Key Laboratory for Mesoscopic Physics and Frontiers Science Center for Nano-optoelectronics, School of Physics, Peking University, Beijing 100871, China

1. Experimental setup and sample characterization

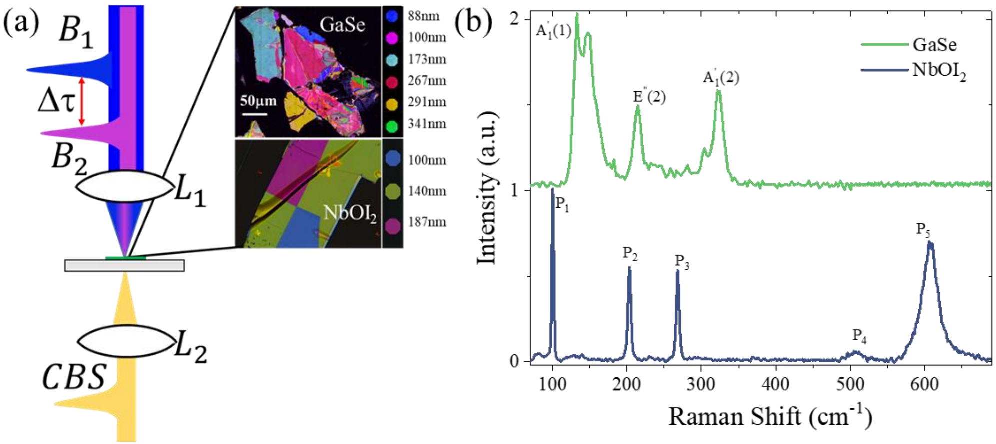

Figure S1: Experimental setup and sample characterization results. (a) Schematic of the experimental setup, optical images of GaSe and $NbOI_2$ flakes on a glass substrate shown in the inset with the corresponding thicknesses at different color regions, (b) Raman spectrum of the GaSe (green line) and $NbOI_2$ (blue line) samples.

The schematic of the experimental setup is shown in Figure S1(a). The optical image of the GaSe and $NbOI_2$ flakes on a glass substrate and the corresponding thickness to different colors are given in the inset. Raman spectrum of the samples, obtained using continuous-wave laser excitation at 532 nm, shows three peaks, $A_1'(1)$, $E''(2)$, and $A_1'(2)$ at ~133 cm^{-1}, ~214 cm^{-1}, and ~322 cm^{-1} respectively for GaSe (Figure S1b top). and five peaks P_1, P_2, P_3, P_4, and P_5 at ~100 cm^{-1}, ~203 cm^{-1}, ~268 cm^{-1}, ~508 cm^{-1}and ~606 cm^{-1} (Figure S1b bottom). Note that the Raman peaks are similar for all thicknesses of GaSe and $NbOI_2$ flakes and agree well with the previously reported results [1, 2], which confirms the high quality of our GaSe and $NbOI_2$ flakes.

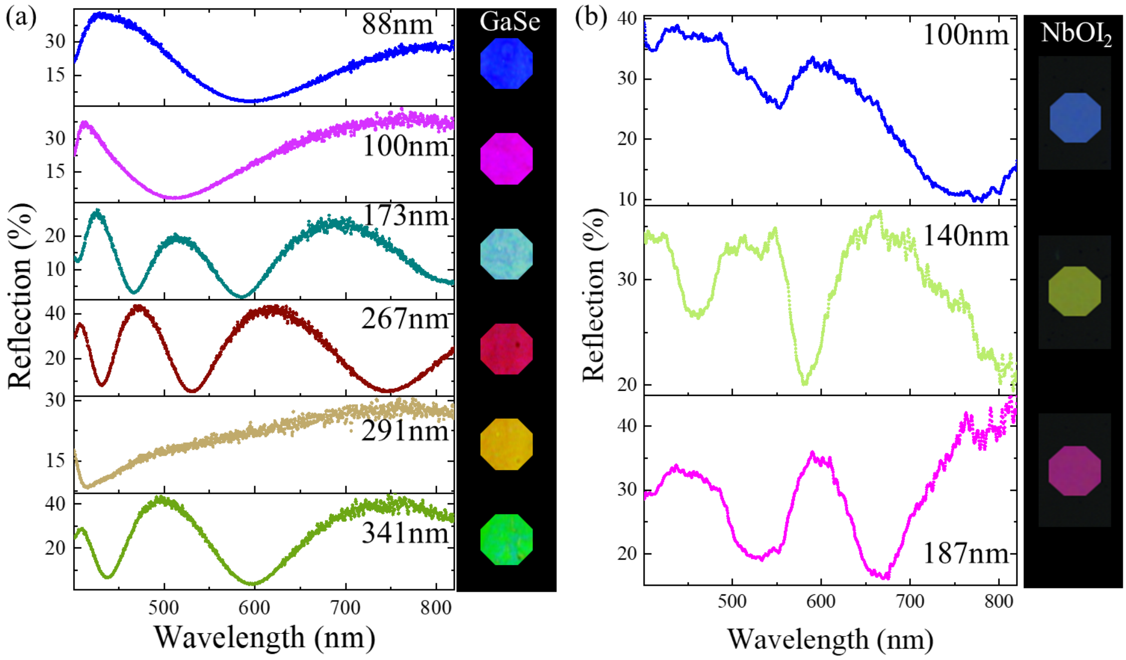

Figure S2: Thickness-dependent reflection spectra of flakes. Thickness-dependent reflected spectra of samples (a) GaSe and (b) $NbOI_2$ flakes on glass substrates.

2. Bandwidth of CBS for different nonlinear processes

Typically, the generated wavelength in the DFG process is given by the equation.

$$\frac{1}{\lambda_{DFG}} = \frac{1}{\lambda_1} - \frac{1}{\lambda_2} \qquad (1)$$

where λ_1 and λ_2 are the central wavelengths of the input beams B_1 and B_2, respectively. Now, we assume that the input beams B_1 and B_2 have the spectral widths of $\Delta\lambda_1$ and $\Delta\lambda_2$, respectively. Consequently, the generated wavelength in the DFG process will also expand. Following the above equation, the longest wavelength (λ_{max}) and the shortest wavelength (λ_{min}) generated by the DFG process can be written as

$$\frac{1}{\lambda_{max}} = \frac{1}{\lambda_1 + \frac{\Delta\lambda_1}{2}} - \frac{1}{\lambda_2 - \frac{\Delta\lambda_2}{2}} \qquad (2)$$

$$\frac{1}{\lambda_{min}} = \frac{1}{\lambda_1 - \frac{\Delta\lambda_1}{2}} - \frac{1}{\lambda_2 + \frac{\Delta\lambda_2}{2}} \qquad (3)$$

where we assume $\lambda_1 < \lambda_2$, and the spectrum of beams B_1 and B_2 are non-overlapping. Therefore, the total bandwidth of the generated DFG signal will be

$$\Delta\lambda = \lambda_{max} - \lambda_{min} = \frac{4\lambda_1^2\Delta\lambda_2 + 4\lambda_2^2\Delta\lambda_1 - \Delta\lambda_1\Delta\lambda_2(\Delta\lambda_1 + \Delta\lambda_2)}{4(\lambda_2 - \lambda_1)^2 - (\Delta\lambda_1 + \Delta\lambda_2)^2} \qquad (4)$$

Indeed, the generated DFG bandwidth depends on the bandwidth of both the beams B1 and B2. Since the spectral width of B1 is very small, for simplification, we assume $\Delta\lambda_1 \approx 0$. Then equation (4) reduces to

$$\Delta\lambda = \frac{4\lambda_1^2\Delta\lambda_2}{4(\lambda_2 - \lambda_1)^2 - \Delta\lambda_2^2} \qquad (5)$$

which is the equation 1 in the main text.

Again, if we assume the spectral width of B2 is negligible, i.e. $\Delta\lambda_2 \approx 0$, then the equation 4 reduced to

$$\Delta\lambda = \frac{4\lambda_2^2\Delta\lambda_1}{4(\lambda_2 - \lambda_1)^2 - \Delta\lambda_1^2} \qquad (6)$$

Comparing Equation 5 and Equation 6, both are very similar in nature. In a similar fashion, the bandwidth of the Four Wave Mixing (FWM) signal can also be calculated. Simulated results for both DFG and FWM are presented in Figure S3 and Figure S4, respectively.

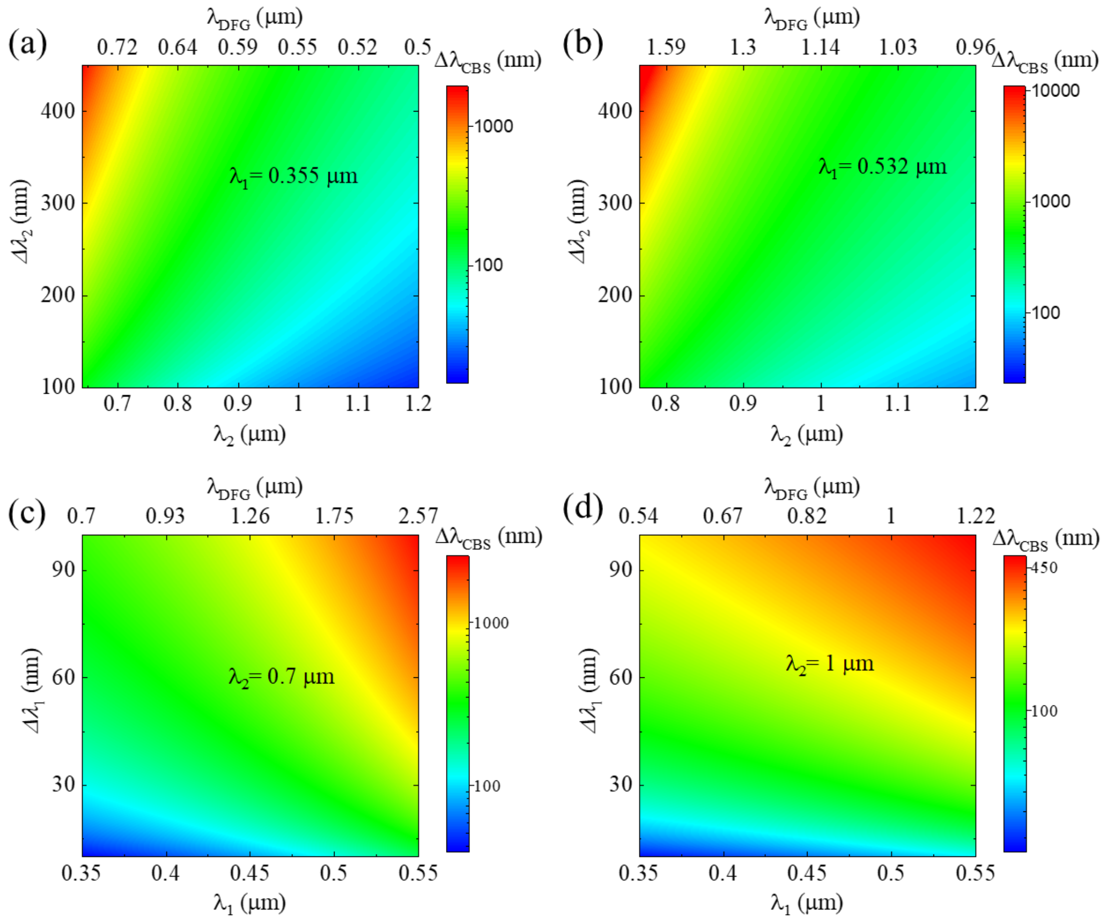

Figure S3: Output spectral width of DFG-based CBS at different pump wavelengths. (a) λ_1 is at 355 nm, (b) λ_1 is at 532 nm, (c) λ_2 is at 700 nm, (d) λ_2 is at 1000 nm.

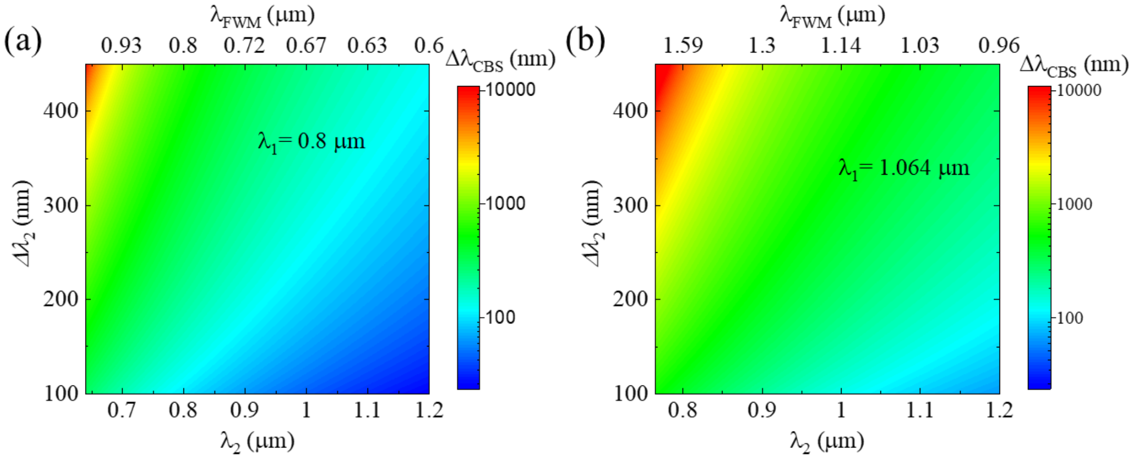

Figure S4: Output CBS spectral width of four-wave mixing, a third-order nonlinear optical process. (a) λ_1 is at 800 nm, (b) λ_1 is at 1064 nm.

3. Coherence of generated wavelength

To verify the state of coherence of the generated signal, we perform a Michelson interferometer experiment, and the schematic is shown in Fig S5(a). The interference fringe is collected by the CCD after passing through a bandpass filter. As the CCD has the highest sensitivity in the visible light range, visible light centered at 650 nm (bandwidth 40 nm) is used to observe the fringe. The fringe on CCD is shown in Fig S5(b) together with the fringe patterns (Figure S5(c)) at different positions indicated by color lines and the corresponding fringe visibility factor (v) calculated as

$$v = \frac{I_{max} - I_{min}}{I_{max} + I_{min}}$$

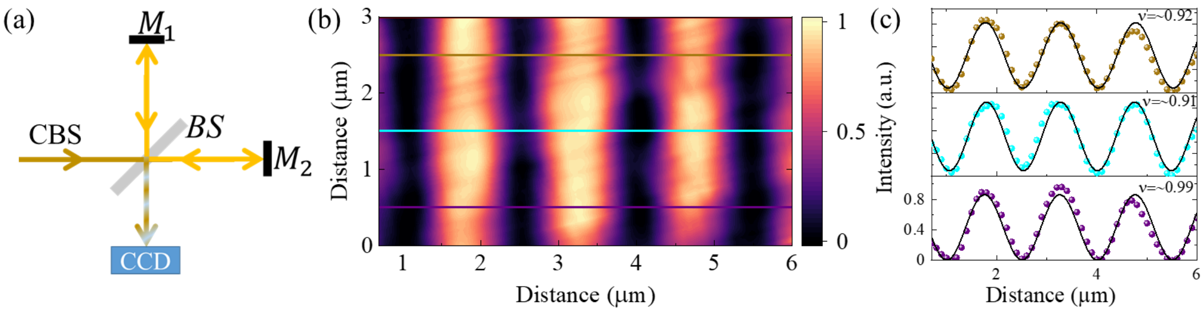

Figure S5: Coherence of the generated light. (a) Schematic of Michelson interferometer experiment. CBS: Our coherent broadband, M: Mirror, BS: Beam splitter, CCD: charge-coupled device, (b) Image of interference fringe on CCD and (c) the fringe patterns at respective positions of color lines with fringe visibility factor (right).

Since we used a 50:50 beam splitter, the intensities of both interfering beams are equal. Therefore, the degree of coherence will be equal to the fringe visibility [3]. The average fringe visibility is ~0.95, which indicates the degree of coherence is very high.

4. Calculation of nonlinear coefficient

The second-order nonlinear optical susceptibility $|\chi^{(2)}|$ for a wide range of wavelengths under different thicknesses of the GaSe flakes is calculated by [4]

$$|\chi^{(2)}| = \frac{4n_s c}{\omega_s}\sqrt{\frac{\varphi f \tau D^2 n_1 n_2 \varepsilon_0 c P_s}{n_s P_1 P_2}}$$

where $\varphi = \frac{1}{8}\left(\frac{\pi}{\ln(2)}\right)^{3/2}$, $\omega_x, P_x,$ and n_x represent the pulse width, frequency, average power, and the refractive index of GaSe with *x=1,2* and *s* define incident beams B_1, B_2 and the DFG signal, respectively. *D* is the beam diameter on the sample. τ, and *f* are the pulse width and repetition rate of the input pulses, respectively; *c* is the speed of light in vacuum, and ε_0 is the vacuum permittivity.

5. CBS spectrum of GaSe

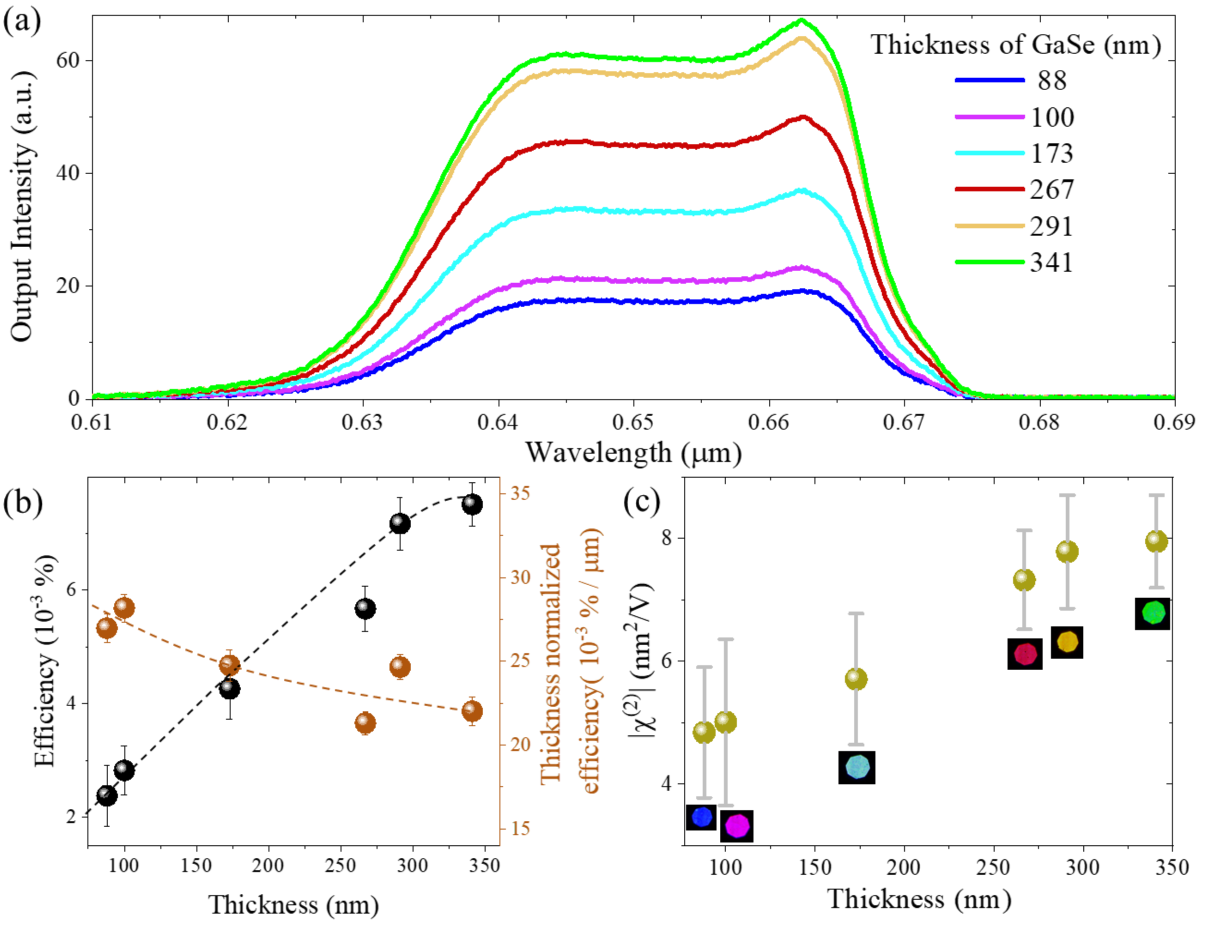

Figure S6: Nanoscale CBS conversion efficiency of GaSe. Thickness-dependent CBS spectra of (a) GaSe under the fixed input powers of ~0.5μW (intensity ~7.67 GW/cm^2) and 5μW (intensity ~76.71 GW/cm^2) for the input beams B_1 and B_2, respectively. (b) Thickness-dependent and thickness-normalized CBS conversion efficiency for GaSe. (c) Calculated thickness-dependent overall $|\chi^{(2)}|$ of GaSe samples. The optical images of the samples at different thicknesses are shown in the inset.

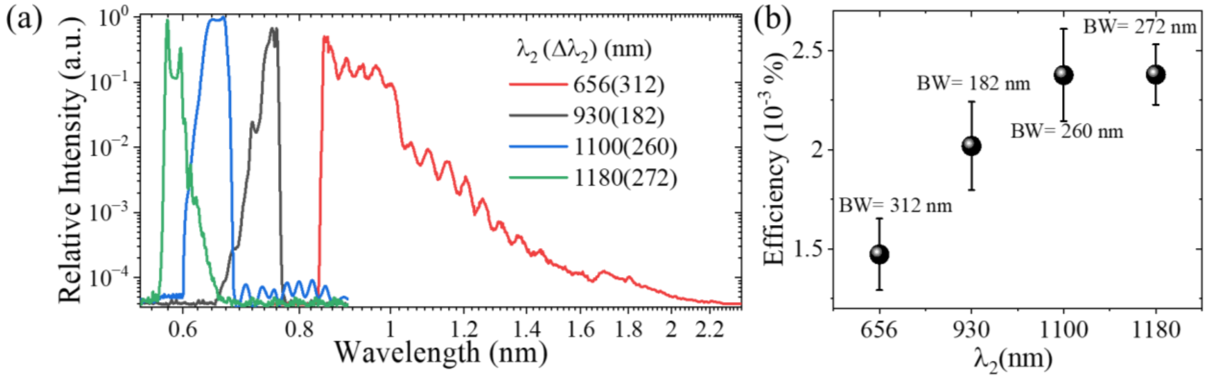

Figure S7: Tunable CBS of GaSe. (a) Relative intensity and (b) calculated efficiency corresponding to incident wavelength.

6. Power dependency of the CBS generation process

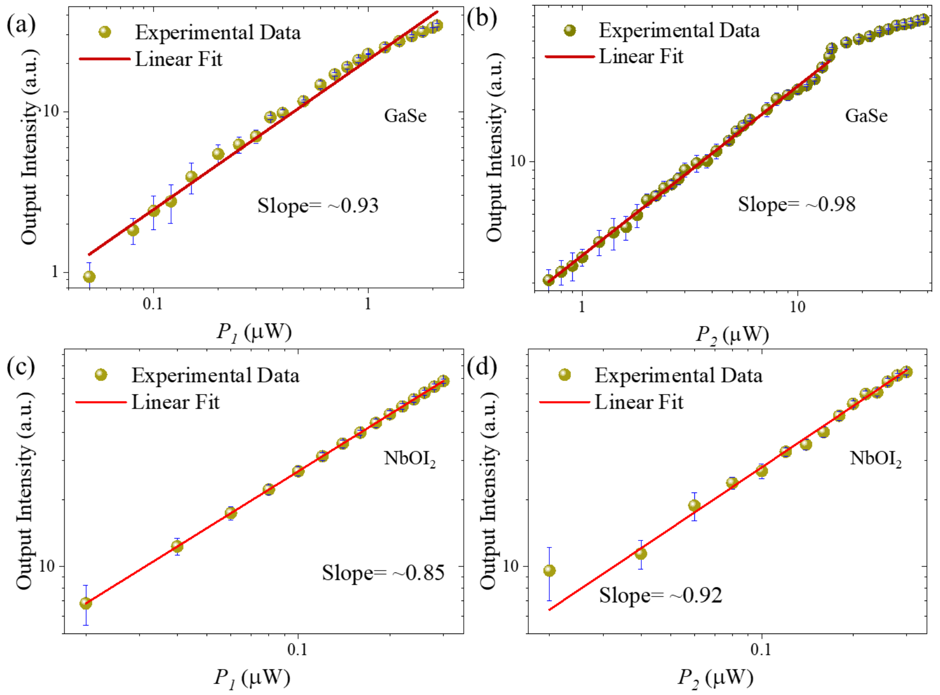

Figure S8: The output power performance of nanoscale CBS generation. (a) The output power of CBS (a) from GaSe flake as a function of the average power of the incident beam B_1 under ~5 μW (intensity ~76.71 GW/cm^2) power of the incident beam B_2, and (b) as a function of the average power of the incident beam B_2 under ~0.5 μW (intensity ~7.67 GW/cm^2) power of the incident beam B_1. (c) from $NbOI_2$ flake as a function of the average power of the incident beam B_1 under ~0.1μW (intensity ~1.53 GW/cm^2) power of the incident beam B_2, and (d) as a function of the average power of the incident beam B_2 under ~0.1μW (intensity ~1.53 GW/cm^2) power of the incident beam B_1.

We observe the linear power pendency of both incident beam powers for both GaSe (Figure S7 (a) and (b)) and $NbOI_2$ (Figure S7 (c) and (d)) flakes. Here, we noticed that the damage threshold for $NbOI_2$ flake (~0.5μW, intensity ~7.67 GW/cm^2) is much lower than the GaSe flakes (~15μW, intensity ~230.13 GW/cm^2).

7. Comparison of critical threshold energy to generate broadband light

The list of critical threshold energy to generate broadband source is provided in the following table for different crystals [5]. In our work, we achieved 2 and 3 orders of magnitude lower critical threshold energy for GaSe and $NbOI_2$ flakes, respectively.

Table 1: Critical energy comparison

Material	Critical energy for broadband generation (nJ)	Interaction Length (μm)	Reference
$NbOI_2$	~0.025	0.1	Our work
GaSe	~0.5	0.088	Our work
YVO_4	~48	4000	Reference 5
$GdVO_4$	~78	4000	Reference 5
KGW	~83	4000	Reference 5
YAG	~172	4000	Reference 5
Sapphire	~350	3000	Reference 5

We also compare our proposed idea with existing literatures on 2D and thin material assisted broadband generation [6-9] and tabulate in Table 2.

Table 2: Comparison of other 2D/thin material assisted broadband generation and our work

Properties	Ref. 6	Ref. 7	Ref. 8	Ref. 9	Our Work
Material / Model	n-InAs Sn-doped	AZO	WS_2-SiN-WG	GO-Si WG	$NbOI_2$ (GaSe)
Operation principle	SOR	SHG + THG + FWM	SPM	SPM	DFG
Interaction Length	100nm - 200nm	140 nm	~14.8 μm	400 μm	< 100nm
Conversion efficiency (output power/total input power)	Estimated ~0.0032%	Estimated ~ 0.016%	-	-	~0.12% (~7.6×10^{-3} %)
Threshold Energy	0.1 mJ	~0.32 μJ	~0.31 nJ	~8.2 pJ	~0.025 (~0.5) nJ
Output bandwidth	0.3 to 0.7 THz	406 to 1100 nm	~672.7 to 900.8 nm	~1546.5 to 1551 nm	Covering 560 to 2000 nm
Coherence	-	Non coherent	-	-	Highly coherent (>0.9)

SOR: Surface Optical Rectification; AZO: Aluminum-doped Zinc Oxide; DFG: Difference Frequency Generation; SPM: Self-Phase Modulation; SHG: Second harmonic generation, THG: Third harmonic generation, FWM: Four wave mixing.

We compare our proposed idea with existing fiber/waveguide-based broadband generation in Table 3.

Table 3: Comparison of other fiber/waveguide-based broadband generation [10-20] and our work

Material / Model	Critical energy for broadband generation (nJ)	Interaction Length (μm)	Reference
$NbOI_2$	~0.025	0.1	Our work
GaSe	~0.5	0.088	Our work
**Fiber Based SCG**			
DF-MF	~0.13	80×10^6	Ref.10
SMF	~5.8	100×10^6	Ref.11
ZBLAN	~0.55	25×10^6	Ref.12
Tapered Fiber	~51.1	$.08\times10^6$	Ref.13
Microstructured Fiber	~0.9	15×10^4	Ref.14
PCF	~1	16×10^4	Ref.15
PCF	~100	1×10^6	Ref.16
**Waveguide Based SCG**			
PPLN	0.038	5000	Ref.17
USRN	0.017	3000	Ref.18
AlGaAs-OI	~0.003	3000	Ref.19
TFLN	~0.015	5000	Ref.20

DF-MF: Dispersion-Flattened Microstructure Fiber; SMF: Single Mode Fiber; ZBLAN: ZrF_4-BaF_2-LaF_3-AlF_3-NaF; PCF: Photonic Crystal Fiber; PPLN: Periodically Poled Lithium Niobate; USRN: Ultra-Silicon-Rich Nitride; TFLN: Thin-Film Lithium Niobate

8. Futuristic integrated application

We have currently designed two possible straightforward ways for integrated integration. (1) The first one is based on an up-reflecting waveguide that can integrate 2D materials like GaSe and $NbOI_2$ on its cross-sectional surface (Fig. S9a). Input light interacts with the 2D materials of the up-reflecting waveguide, and the generated broadband will be guided by the waveguide before exiting the up-reflecting waveguide through the output port. (2) The second one is based on a typical waveguide where 2D materials like GaSe and $NbOI_2$ are integrated on its cross-sectional surface (Fig. S9b)[21]. Input light guided by the waveguide interacts with the 2D materials of the integrated waveguide and the generated broadband will be guided by the waveguide before exiting through the output port. By using the waveguides solely as a guided medium for light, rather than being an active medium for nonlinear phenomena, the phase-matching constraint can be more easily avoided. In this design, it is crucial to position 2D materials in close proximity to the input port to mitigate issues related to dispersion.

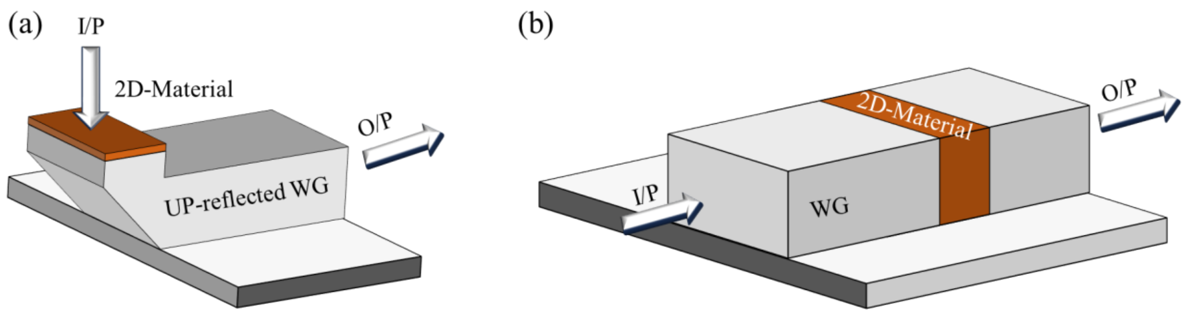

Figure S9: Schematic of integrated CBS generation by integrating 2D-materials with (a) up-reflecting silicon nitride waveguide, and (b) typical silicon nitride waveguide. I/P: input port; O/P: output port; WG: waveguide. I/P: input port; O/P: output port; WG: waveguide."

References

1. Diep, N.Q. et al. Screw-Dislocation-Driven Growth Mode in Two Dimensional GaSe on GaAs(001) Substrates Grown by Molecular Beam Epitaxy. *Scientific Reports* **9**, 17781 (2019).
2. Fang, Y., Wang, F., Wang, R., Zhai, T. & Huang, F. 2D NbOI2: A Chiral Semiconductor with Highly In-Plane Anisotropic Electrical and Optical Properties. *Advanced Materials* **33**, 2101505 (2021).
3. Biswas, T., García Díaz, M. & Winter, A. Interferometric visibility and coherence. *Proceedings of the Royal Society A: Mathematical, Physical and Engineering Sciences* **473**, 20170170 (2017).
4. Dai, Y. et al. Electrical control of interband resonant nonlinear optics in monolayer MoS_2. *ACS nano* **14**, 8442-8448 (2020).
5. Bradler, M., Baum, P. & Riedle, E. Femtosecond continuum generation in bulk laser host materials with sub-μJ pump pulses. *Applied Physics B* **97**, 561-574 (2009).
6. Peters, L., Tunesi, J., Pasquazi, A. & Peccianti, M. High-energy terahertz surface optical rectification. *Nano Energy* **46**, 128-132 (2018).
7. Tian, W. et al. Highly Efficient Super-Continuum Generation on an Epsilon-Near-Zero Surface. *ACS Omega* **5**, 2458-2464 (2020).
8. Wang, Y. et al. Enhancing Si3N4 waveguide nonlinearity with heterogeneous integration of few-layer WS2. *ACS photonics* **8**, 2713-2721 (2021).
9. Zhang, Y. et al. Enhanced Kerr nonlinearity and nonlinear figure of merit in silicon nanowires integrated with 2D graphene oxide films. *ACS Applied Materials & Interfaces* **12**, 33094-33103 (2020).
10. Yong-Zhao, X., Xiao-Min, R., Zi-Nan, W., Xia, Z. & Yong-Qing, H. Flat supercontinuum generation at 1550 nm in a dispersion-flattened microstructure fibre using picosecond pulse. *Chinese Physics Letters* **24**, 734 (2007).
11. Husin, S.A.S. et al. Narrow core standard single mode fiber for supercontinuum generation from graphene-based mode-locked pulses. *Optik* **172**, 347-352 (2018).
12. Yemineni, S.R., Lai, W.J., Alphones, A. & Shum, P. Mid-IR supercontinuum generation in a single-mode ZBLAN fiber by erbium-doped fiber laser. *Optical Engineering* **57**, 111804-111804 (2018).
13. Ahmad, H., Salleh, M., Zaini, M.K.A., Samion, M.Z. & Reduan, S.A. Performance comparison of supercontinuum generation using various tapered fiber waist diameters with a mode-locked fiber laser. *Optik* **272**, 170414 (2023).
14. Corwin, K.L. et al. Fundamental noise limitations to supercontinuum generation in microstructure fiber. *Physical review letters* **90**, 113904 (2003).
15. Dudley, J.M. et al. Cross-correlation frequency resolved optical gating analysis of broadband continuum generation in photonic crystal fiber: simulations and experiments. *Optics Express* **10**, 1215-1221 (2002).
16. Hooper, L.E., Mosley, P.J., Muir, A.C., Wadsworth, W.J. & Knight, J.C. Coherent supercontinuum generation in photonic crystal fiber with all-normal group velocity dispersion. *Optics express* **19**, 4902-4907 (2011).
17. Yu, M., Desiatov, B., Okawachi, Y., Gaeta, A.L. & Lončar, M. Coherent two-octave-spanning supercontinuum generation in lithium-niobate waveguides. *Opt. Lett.* **44**, 1222-1225 (2019).
18. Cao, Y. et al. Supercontinuum generation in a nonlinear ultra-silicon-rich nitride waveguide. *Scientific Reports* **12**, 9487 (2022).
19. May, S., Clerici, M. & Sorel, M. Supercontinuum generation in dispersion engineered AlGaAs-on-insulator waveguides. *Scientific Reports* **11**, 2052 (2021).
20. Hamrouni, M. et al. Picojoule-level supercontinuum generation in thin-film lithium niobate on sapphire. *Optics Express* **32**, 12004-12011 (2024).
21. Cui, X. et al. On-chip photonics and optoelectronics with a van der Waals material dielectric platform. *Nanoscale* **14**, 9459-9465 (2022).
